# Supplementary material for: The Specific ROCK2 Inhibitor KD025 Alleviates Glycolysis through Modulating STAT3-, CSTA- and S1PR3-Linked Signaling in Human Trabecular Meshwork Cells
Source: Biomedicines. 2024 May 24;12(6):1165. doi: 10.3390/biomedicines12061165 (PMC11200618; doi:10.3390/biomedicines12061165)
Supplement: Supplementary file 1 [file biomedicines-12-01165-s001.zip › A_vs_B_DEG_MA_plot_padj.html]

A\_vs\_B\_DEG\_MA\_plot\_padj
